# Anthroposomics: integrating anthropological methods into exposome research

**DOI:** 10.1186/s12940-025-01225-z

**Published:** 2025-10-14

**Authors:** Anita Hardon, Martha M. Téllez-Rojo, Michael Anastario, Michael Lim Tan, Cecilia S. Alcala, Precious A. Echague, Amy Kuritzky, Talia R. Gordon, Zoe Boudart, Mariana Rios Sandoval, Elizabeth F.S. Roberts

**Affiliations:** 1 Knowledge Technology and Innovation Chair group, Wageningen University and Research, Wageningen, Gelderland the Netherlands; 2 Instituto Nacional de Salud Pública, Universidad No. 655 Colonia Santa María Ahuacatitlán, Cuernavaca, Morelos México; 3 Department of Health Sciences, Northern Arizona University, Flagstaff, AZ USA; 4 Department of Anthropology, University of the Philippines Diliman, Quezon City, The Philippines; 5 Department of Environmental Medicine, Icahn School of Medicine at Mount Sinai, New York, NY USA; 6 Department of Anthropology, University of Michigan, Ann Arbor, MI USA

**Keywords:** Exposome, Ethnography, Methods, Anthroposomics, Community health interventions

## Abstract

**Background:**

Exposome research seeks to understand how cumulative environmental exposures across the life course shape health outcomes. Most studies however, adopt a unidirectional, top-down model, conceptualizing individuals as passive recipients of exposure, which overlooks the social, cultural, and behavioral dynamics through which people engage with their environments and thus underestimates the human agency of those exposed in mitigating exposures.

**Main body:**

To address this gap, we introduce the concept of the *anthroposome*: the full range of micro-ecological practices through which individuals and communities sense, interpret, avoid, and manage environmental exposures in daily life. Drawing on anthropological theory and focusing on ethnographic methods, we outline five discovery-based approaches for integrating lived experience and social complexity into exposome science. These methods highlight how everyday practices influence exposure pathways and reveal context-specific risk management strategies conventional exposure researchers may miss. Capturing bidirectional human–environment interactions, anthroposomics repositions populations suffering from exposure as active agents who participate in shaping their exposure landscapes.

**Conclusion:**

Anthroposomics expands the exposome paradigm by integrating ethnographic methods into exposome research, enhancing the paradigm's relevance, effectiveness, and equity. Anthroposomics offers a foundation for preventive, community-responsive, justice-oriented environmental health interventions and policy.

## Background

In 2005, International Agency for Research on Cancer Director Christopher Wild proposed the “exposome” as a term to describe the totality of environmental exposures a person faces over a lifetime, essentially an environmental complement to the genome. Wild's vision for exposome research involved interdisciplinary collaboration among the social, life, and natural sciences to help unravel the complexity of the exposome [1, 2]. Since then, scientists have gone on to characterize general and specific external exposomes using advanced sensing technologies [3–8] and applied sophisticated “omics” to enhance our understanding of internal biological processes and the pathways that mediate external exposures and shape health outcomes [9–11]. Employing life and applied sciences technologies, tools, and methods has expanded our understandings of the general and specific exposures that shape human health and the biological mechanisms through which they do so. Exposome researchers now argue that their research data can refine precision medicine by providing evidence of corporeal responses to environmental exposures and improve diagnoses and treatment of individual patients in clinical settings [10].

In the meantime, a number of social scientists, public health experts, and environmental justice scholars have called for integrating social and population level variables into exposome research [12–15] to elucidate how structural variables stratify adverse health outcomes. Notably, attendees at a recent NIHES workshop stressed the importance of community-generated exposomics to improve interventions, indicating that traditional toxicology fails to address the vast diversity in people’s daily exposures [16]. This commentary advocates for further integrating social dynamics into exposome research by including discovery-oriented anthropological approaches that contribute additional insights into how environments affect health in communities, which can inform community-level mitigation efforts.

**Fig. 1:**
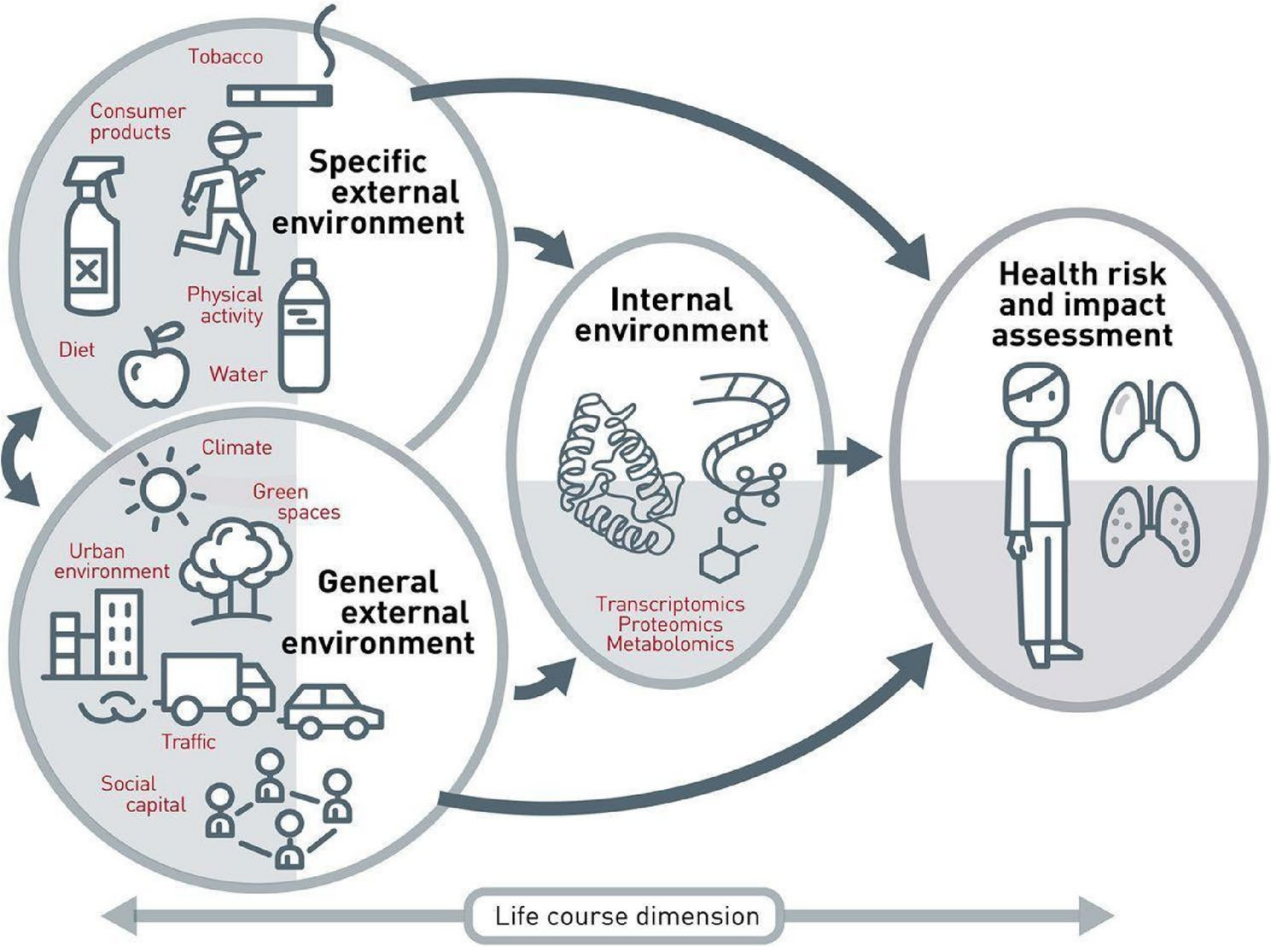
The Еxposome [17]

Despite calls for community exposomics that incorporate social analyses, the bulk of exposome research still conceptualizes individuals as passive recipients of exposures, rather than as people with agency in shaping and mitigating them [4–6]. Figure 1 presents a typical depiction of the exposome derived from “The Exposome: A New Paradigm to Study the Impact of the Environment on Health.” [17]. Its arrows illustrate exposures moving from general, specific external, and internal environments affecting a static individual person.

Such exposome models inadvertently reinforce epistemo-methodological assumptions that obscure significant socioenvironmental relations and everyday practices—including those engaged in by people seeking to mitigate harm—which shape exposures and health outcomes. Arrows uniformly moving towards a single passive person reinforce individualized approaches that seem best treated by clinical medicine, discouraging structural measures, or consideration of collective activism by people affected by exposures. Moreover, the implicit “arrow-in” model overlooks an entire set of situated, dynamic, bidirectional human–environment interactions and micro-ecological practices people in affected communities (individually and collectively) employ to sense, know, avoid, mitigate, and transform exposures every day and over their lifespans. We call these practices the *anthroposome* and the study of them *anthroposomics*.

We acknowledge that social and public health exposome studies [14] go beyond the individual by examining how social variables such as ethnicity, education, income, and housing conditions shape inequality in the social patterning of risks and exposures [16, 18, 19]. However, these studies tend to focus on known variables, thus reinforcing “the arrow in” by relying on deductive analytic frameworks that operationalize community data and social variables as fixed predictors of health outcomes. While critical to elucidating structural determinants and community-level exposures, such variables are contingent on the everyday micro-ecological practices that shape and mediate exposures. Understanding the anthroposome requires systematic examination of data generated using open-ended ethnographic methods and inductive, discovery-based approaches that capture complex, bidirectional dynamics to design interventions in collaboration with affected communities to reduce exposures.

Below, we describe how anthroposomics can play a key role in illuminating the real-life dynamics that shape community exposures and health outcomes and refine the measurement thereof. Employing robust tools that provide fine-grained insights into the *complex*,* not-yet-discovered interplay* between the environment and people’s daily lives, anthroposomics strengthens exposome research by focusing on the micro-ecological practices that shape relations between exposures and health. Broad inclusion of anthropsomics in exposome research would help deepen and integrate discovery-oriented research across existing disciplinary silos [9]. Anthroposomics not only generates qualitative understandings of the situated dynamics that cause differential health outcomes, it also produces a more situated understandings of exposure dynamics, generating quantitative variables that help identify patterns in relation to other omics, insitu.

**Fig. 2:**
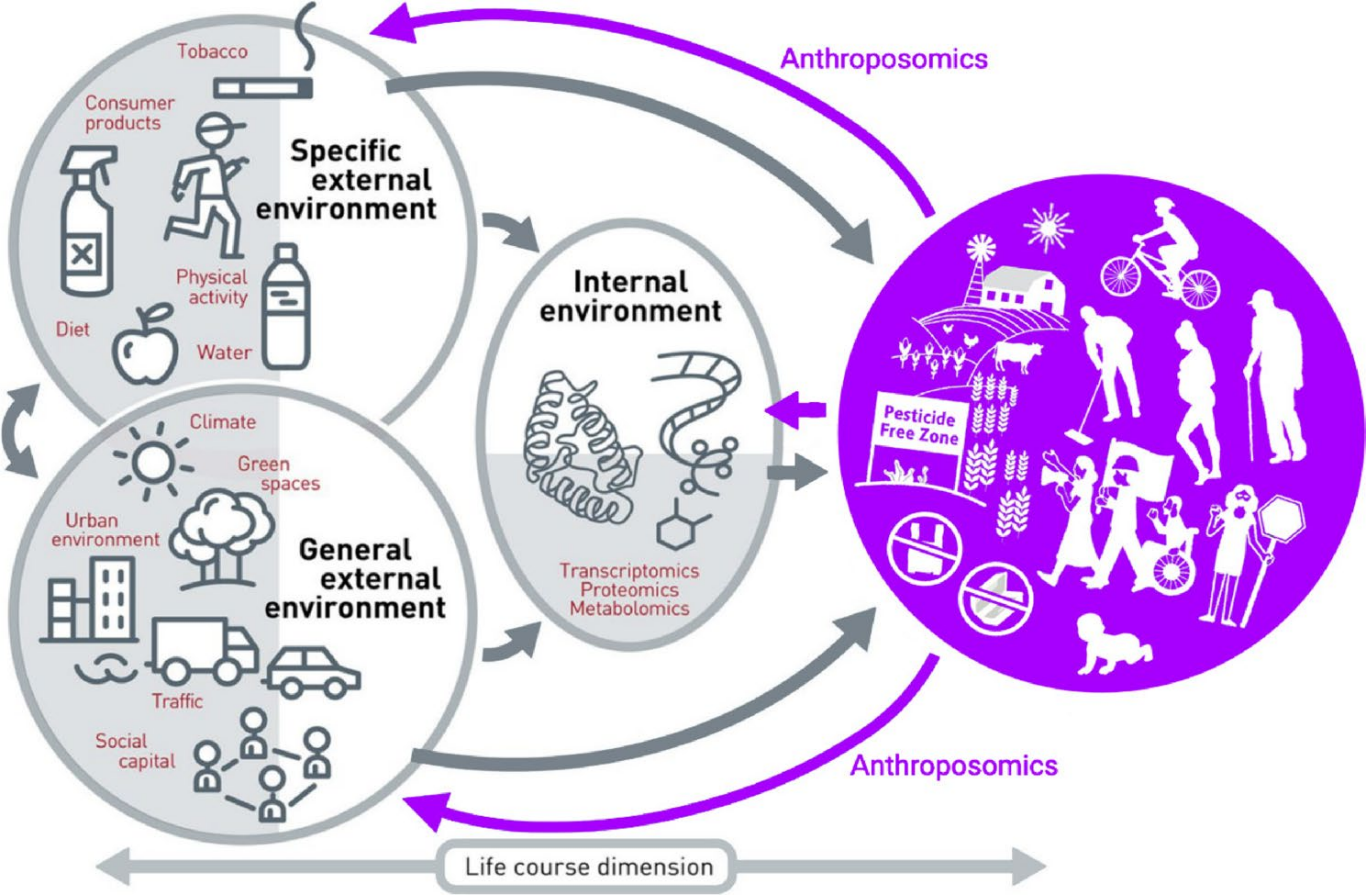
The arrows back

To demonstrate how anthroposomics contributes to exposome research, we reconceived Fig. 1 with multiple people actively shaping the conditions of their everyday lives by engaging in a range of practices. To do this we add “arrows back” to represent contingent, nonlinear, recursive relations with environmental factors. Figure 2 can act as a guide for anthroposomic inquiries. We note that the arrow at the bottom of Fig. 1 above appears to refer to bidirectionality—the life course dimension—but these seemingly indicate time, not to people taking actions to mitigate harm.

Figure 1 includes the actions of people affected by exposures as predefined, individual variables (diet, physical activity, smoking) in specific environments. By contrast, Fig. 2 includes interactions between people and their environments broadly. Anthroposomics does not take as its starting point researchers’ hypotheses regarding which behaviors among exposed people matter, but rather entails goal-free observations of individual and community-wide activities shaping exposures and health outcomes. It replaces the passive individual body affected by specific and general environmental conditions with a purple bubble indicating people of various ages living together in communities of care, in which they collectively experience their environments, collaboratively make sense of their bodily responses to exposures, and take measures to reduce risks or to change their circumstances, all within the constraints of the opportunities afforded to them.

The anthroposomics toolkit we describe below includes five open-ended, inductive methods that contribute to comprehending the complex interplay of environmental, biological, and social factors implicated in the exposome. It illustrates the utility of apprehending the micro-ecological practices that shape communities’ exposures in terms of generating actionable insights to reduce environmental exposure in people’s daily lives and improve health outcomes [20]. We highlight how our methods identify variables for integration into multi-omics research datasets, thereby improving comprehension of the exposome.

### Anthroposomics toolkit

Five methods have proven particularly useful in our own research on exposures in everyday life among a wide variety of communities: (1) open-ended participant observation, (2) life history calendars, (3) experiential/sensory mapping, (4) head-to-toe interviews, and (5) household chemical assessments.

#### 1) open-ended participant observation

Open-ended participant observation is an ethnographic research method commonly employed in anthropological studies to gather data about people, processes, and phenomena in specific sites or contexts [21]. In exposure research, this method can generate fine-grained data about human–environment relations. In contrast to surveys or focus groups, which pose predetermined questions about specific variables or known risk factors, participant observation is a discovery-based approach allowing researchers to study people in their local structural, social, cultural, economic, political, and historical contexts, to understand the complex interplay of factors and forces that shape their exposures over time.

For example, in 2014–2015, Roberts (last author) conducted participant observation in Mexico City over 18 months with families enrolled in Early Life Exposures in Mexico to Environmental Toxicants (ELEMENT), a longitudinal birth cohort study investigating the developmental effects of exposure [22]. US and Mexican environmental health researchers established ELEMENT in the early 1990s in response to the discovery of high lead exposure levels among Mexico City residents. The researchers directing ELEMENT, including Téllez-Rojo (Second Author), found high lead concentrations in participants’ bone and blood samples associated with cooking with and eating off lead-glazed ceramic dishes, *trastes de barro* [23]. When Roberts began ethnographic fieldwork with ELEMENT families, they had been educated to avoid *trastes de barro* for almost two decades.

**Fig. 3:**
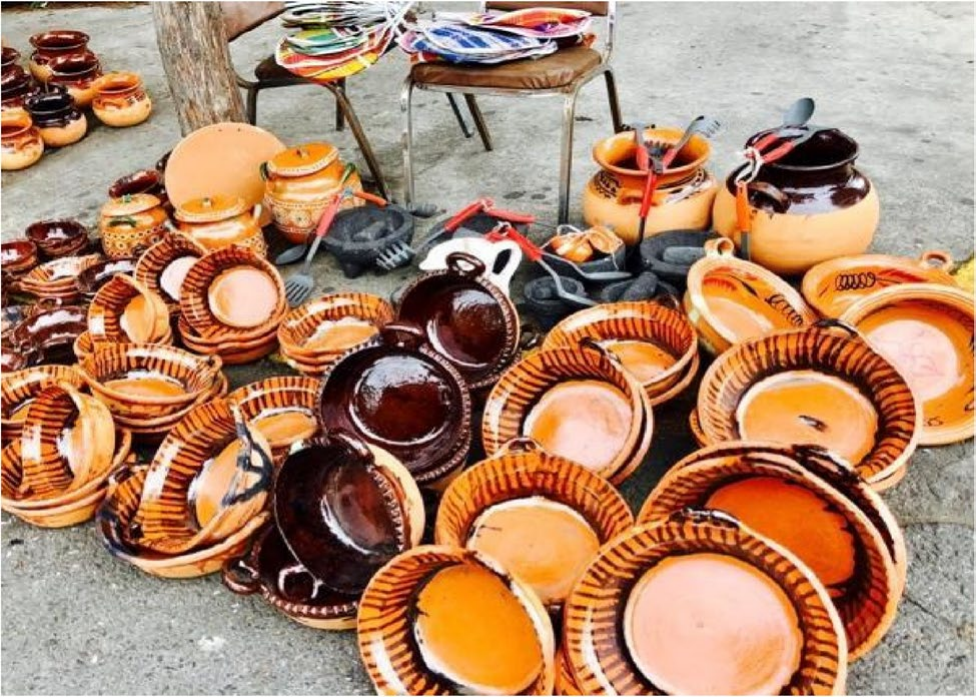
Lead-glazed *trastes de barro* for sale in Mexico City (Photograph by Elizabeth Roberts 2018)

Roberts observed that, despite the warnings, many families continued to use *trastes de barro* (Fig. 3). As part of their families for generations, connecting loved ones at daily meals and celebrations, the lead-glazed dishes shone brightly and imparted sweetness to cooked food. When Roberts’ spent time with families during meals and celebrations, people wondered aloud why ELEMENT researchers focused so intensely on the harmful effects of the dishes while seeming to ignore the ever-present pollution of Mexico City. As one participant told Roberts, “Look at the life we have: the bad air, the pollution. But of course, *they* say we must stop cooking with *trastes de barro*.”

Roberts came to understand that using *trastes de barro* made sense to these families, because they valued the connective power of the dishes more than preventing the risk of lead exposure, which seemed distant from their lives. These insights add an “arrow back” to the ELEMENT project’s exposome research, explaining *why* people continue to use the dishes, despite health education efforts that emphasize lead’s risks. Participating in family gatherings over time taught Roberts the dishes’ importance, particularly for celebrations. Moreover, she witnessed the harm-reduction strategies families developed to keep using the dishes. Some families limited their use to a few times a year and avoided using the dishes to serve acidic foods, thus somewhat mitigating their harm.

Our ethnographic findings regarding how and why participants continue using the dishes could be adapted for environmental health intervention strategies. ELEMENT researchers understand their efforts to convince participants to stop using unleaded glazes may be more complicated than initially thought because the lead imparts qualities that the families value. Informed by anthropological insights, ELEMENT researchers are developing a largescale intervention aimed at reducing lead exposure that consider local conditions, including frequency of use, use with acidic foods, and participants’ attachments to *trastes de barro*. The intervention will be co-designed with a non-governmental childhood development organization, *Un Kilo de Ayuda*, the communities, and our team. We are planning to include an anthropologist with extensive experience working with lead-glazed ceramics in rural and indigenous communities, to bring the arrow back in the intervention design.

#### 2) life history calendars

**Fig. 4:**
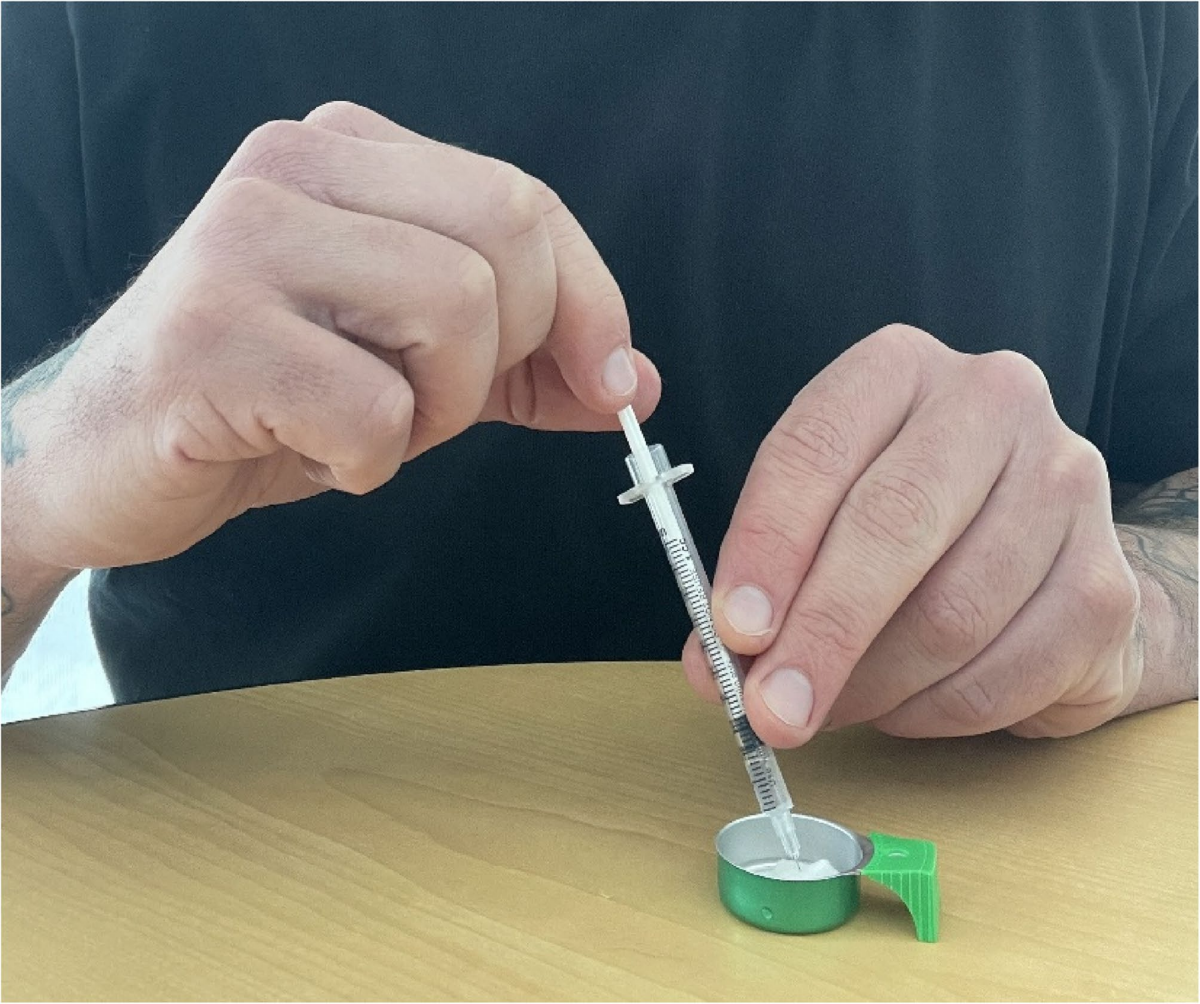
Demonstrating a cigarette butt filtering a methamphetamine preparation (Photograph by Michael Anastario June 2025)

In his research with Native Americans in the United States who inject methamphetamine, Anastario (3rd author) created life history calendars (LHCs) to understand complex histories of polysubstance use. LHCs improve recall by aligning recorded life events with autobiographical memory [24, 25]. Anastario adapted LHC's to measure polysubstance use sequences across lifespans, training LHC administrators to observe, take field notes on the instrument, and document the open-ended comments made during semi-structured interviews that facilitated the LHCs. During LHCs, participants referenced using makeshift filters—cotton, Q-tip ends, cigarette butts, and paper towels—to filter out impurities from methamphetamine preparations as they are drawn into the syringe barrel prior to injecting them (Fig. 4).

Participants asked LHC interviewers whether these filters remove the “battery acid”, “cloudy meth”, and “all that shit” that contaminates methamphetamine preparations, which revealed that users had health concerns about the contents of their preparations 

[26]. Anastario investigated further and indeed found a range of toxicants potentially contaminating methamphetamine injection preparations, such as compounds linked to copper chloride, lead acetate, and mercury used in methamphetamine production [27, 28]. Injecting these metals can potentially lead to multiple organ damage and neurotoxicity and exacerbate infections and cardiovascular disease.

**Fig. 5:**
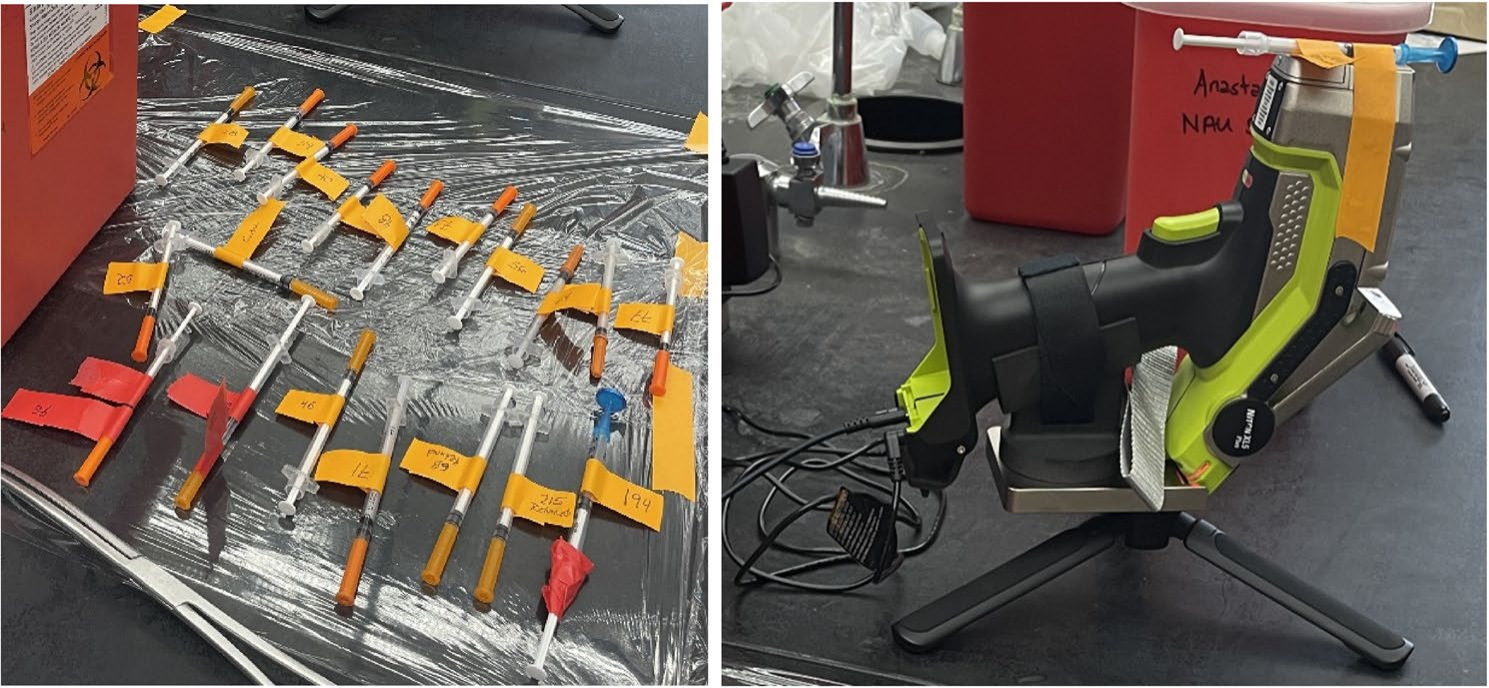
Left: Syringes Anastario tested for metals. Right: handheld pXRF device used in study. (Photograph by Michael Anastario June 2025)

Anastario asked research participants if they would be willing to provide biosamples. While many were unwilling to provide typical biospecimens (blood, urine, hair), some were willing to exchange a used syringe for new ones [29]. This led to follow-up research where a medical physicist and environmental toxicologist employed field notes, questionnaires, biometric measures, and portable X-ray fluorescence (pXRF) to detect metals in used syringes that participants provided (Fig. 5).

The research team detected metals (Zn, Ni, As, Hg) in both used syringes and seized methamphetamine samples. The practice of filtration, articulated through ethnographic methods (LHCs) could be quantified and represented with a binary variable (0 = does not regularly filter preparation, 1 = filters preparation) for the sample. This ethnographically generated variable was then entered as an independent variable in a negative binominal regression, which was used to model the count of metals detected in the syringe barrels, controlling for potential confounders including whether the syringe was washed by the participant prior to arriving to the study site, the number of times the syringe was used, and whether the participant lived with a construction, industrial, or oil worker. The team found that after adjusting for potential confounders, the practice of filtering the injection preparation was associated with a reduced number of metals detected in the syringe (adjusted incident rate ratio = 0.20, 95% CI 0.06–0.72) [28]. In this case, the ethnographically identified filtration variable that was included in the negative binomial regression is a *practice* as it reflects an action situated with a logic that filtering injected meth probably removes metals. Ethnographic methods allowed for the discovery and subsequent quantification of this practice (not known to researchers) that exists within networks of people who inject drugs. The quantification of the practice of filtering methamphetamine is an example of how anthroposomics can identify exposure modifications that could be easily missed in other omics, which also exhibits a capacity to generate data amenable to quantitative modeling.

#### 3) sensory mapping

While exposome researchers rely on a sophisticated array of satellites, sensors, and wearable devices to measure air quality in relation to health outcomes in various locations, ethnographic sensory mapping can reveal dynamics that neither satellite data nor pollution-measuring devices can capture. Sensory maps depict people’s embodied, sensorial relations with space (urban, domestic, or working spaces, to name but a few). This kind of cartography can be carried out by researchers or research participants, who can directly represent, through drawing, collaging or other techniques, how they inhabit and navigate daily exposure. Sensory mapping makes it possible to map how community members and workers in high risk occupations perceive and experience their daily lived environments and how they seek to reduce harm [30, 31].

Hardon’s team (first author) employed sensory mapping with urban dwellers in the Philippines to generate novel insights about air quality that are significant to study participants. Researchers asked participants to produce drawings, collages, illustrations, and other visualizations of their spatialized life histories, showing where they were born, grew up, attended school, worked, and had their children. Instead of inquiring into specific exposures across participants’ lifetimes, researchers asked questions like: “Where do/did you feel good and bad?” Most people reported a sense of well-being in places with vegetation and fresh air, often speaking with nostalgia about the freshness of the air where they grew up, and where their relatives still live. These narratives and the sensorial maps representing them provide thick description of exposome research’s arrow back by focusing not only on individual exposures, but also on how people manage them and what matters to people.

**Fig. 6:**
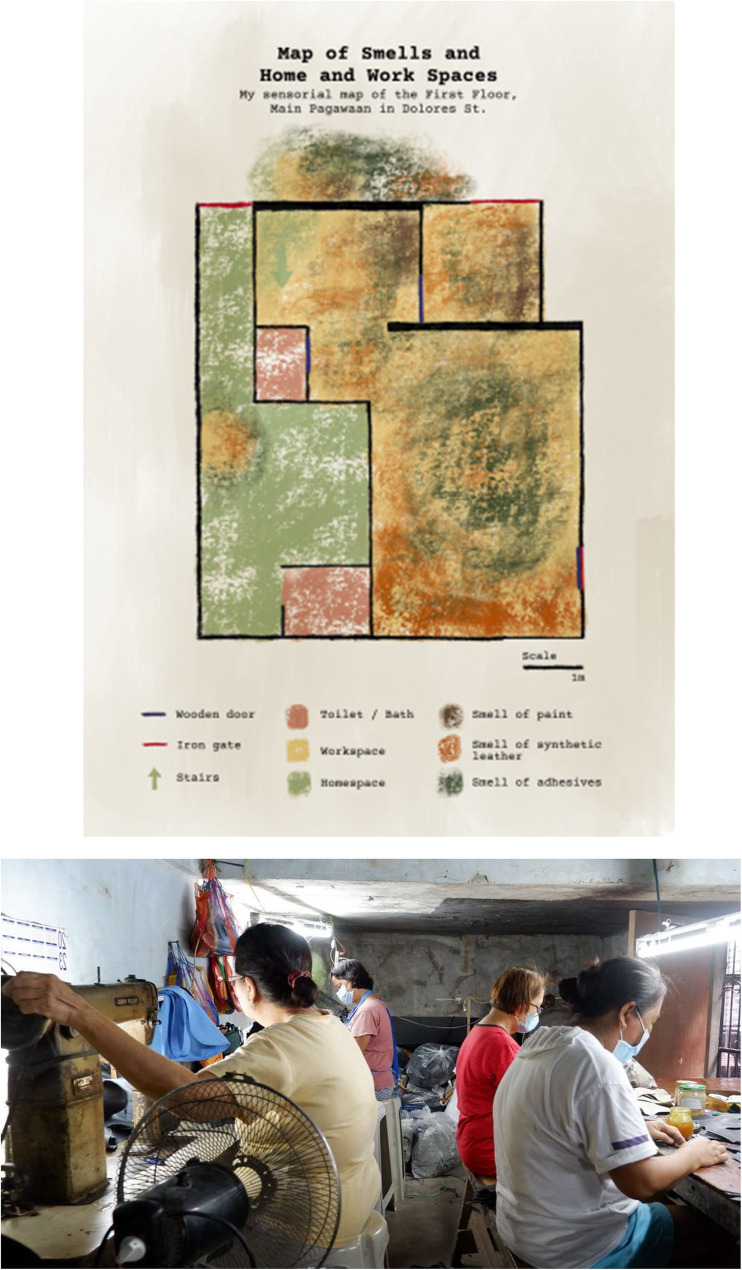
Above: sensory map of Dolores Street, Marikina, the Philippines; Below: Inside view of the shoemaking workshop (Map and Photograph by Precious Echague, October 2024)

Sensory mapping thus provides a layer of specificity regarding pollution as a lived experience to the big data maps that exposome researchers develop to describe the spatiotemporal dynamics of exposure, representing a technique for both researchers and study participants to learn about key exposures in everyday life. Beyond identifying hotspots in the general external environment scientists using non-human sensing methods may miss, sensory mapping generates data on the activities occurring in hotspots that exacerbate air pollution, increasing the potential for precise interventions targeted at reducing exposures. Figure 6 shows a sensory map Precious Echague developed together with residents and workers on Marikina’s Dolores Street—one of Hardon’s fieldsites—where shoemaking is a common way to earn a living. It shows the different kinds of smells (adhesives, paint and leather) that the residents and workers experience in different spaces, including the alley that runs along the workshop.

The map shows the many activities taking place on a narrow street housing two shoemaking workshops, while the photograph, depicts the activities taking place inside the shoemaking workshop, including sewing and gluing and ventilating.

**Fig. 7:**
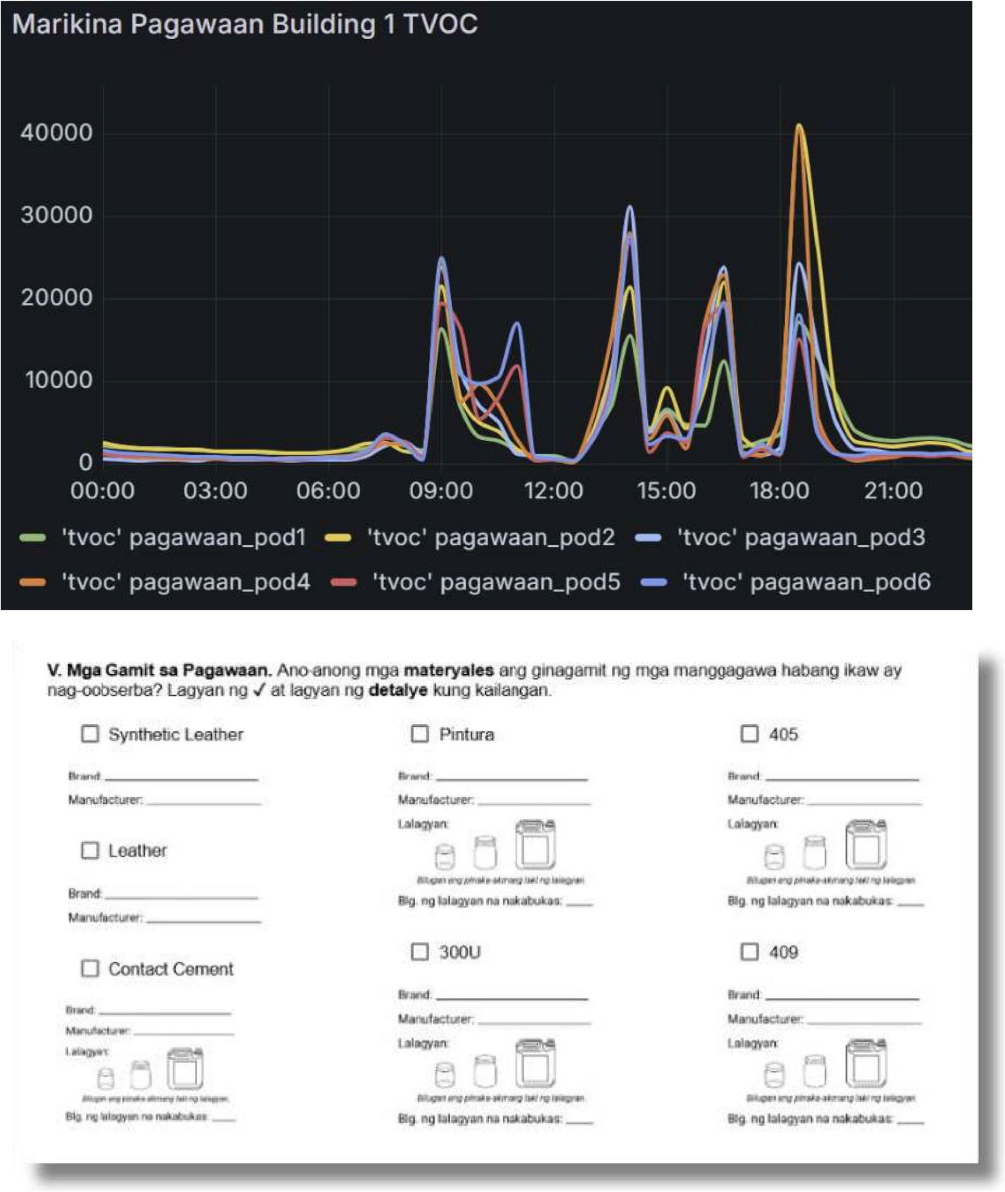
Above: VOC (ppb) levels in a workshop measured by six devices from midnight until 9pm; Below: Part of an observation guide, used to record of workshop gluing and painting practices (Photography by Precious Echague 2025)

During the map-making process, the workshop owner and the workers became interested in further examining the presence of volatile organic compounds (VOCs) in their workplace and exploring ways to reduce risk. Hardon’s team then facilitated exchanges with engineers and airflow modelers to generate a situated understanding of VOC exposures in the workshop (Fig. 7). Together with the workers, Echague helped identify where to place air sensors and the modelers identified ways to improve ventilation to reduce exposures.

Comparing recorded practices (including kinds of glues, leathers and paints used and ventilation practices) with VOC levels allowed us to formulate hypotheses regarding how workshop activities affect exposures. The graphs show VOC levels decreasing during the noon lunchbreak, when the workers stop working and open the door to the alley.

Subsequently, we systematically examined temporal changes in the average total volatile organic compound (TVOC) levels over a single day using time series regression. We used ethnographic data to identify the time intervals when the lunch break occurred and generated a variable in the time series to indicate when workers were observed as being on their lunch break (1) or not (0). We regressed untransformed TVOC levels on the ethnographically generated binary indicator for the lunch period. The residuals from this model exhibited evidence of first-order autocorrelation, as indicated by a Durbin–Watson statistic of 1.39 and significant autocorrelation at lag 1 (AC₁ = 0.30, *p* = 0.033). The distribution of TVOC levels was right-skewed, so we applied a natural logarithmic transformation. To address autocorrelation, we fit a Prais–Winsten regression model with AR(1) correction using the log-transformed outcome. This model yielded a statistically significant decrease in VOC levels during the ethnographically observed lunch time period (β = − 2.18, *p* = 0.001).

Further analysis will examine correlations between container sizes, glue type, ventilation, idling motorcycles in the alley, humidity and outside temperature and VOC levels. This ongoing interdisciplinary research combines observations, static sensing devices, and airflow modelling into a multimodal platform that provides workers with feedback on the impact of future interventions (such as better positioning ventilators and opening doors) on air quality. This specific analysis made clear to the workers that VOC levels fluctuate and that their actions can help reduce levels substantially. We are now working with the shoemakers to also analyze the data from flow modelling to redesign their workshop in order to reduce VOC exposures. Other possible interventions include; adding an exhaust pipe, introducing an air filter, and re-arranging the workspaces, so that workers are placed in the part of the workspace where VOC levels can be reduced through ventilation. When Hardon’s team and the workers deliberated together it became clear that the workers are not in favor of ventilating their worktables while they are gluing because the glue dries too fast impacting the quality of the shoes, nor do they want to rearrange the work stations to face the wall to avoid high VOC levels. Facing each other while working and talking is key to making the time pass while carrying out this labor.

Here too, our anthroposomics approach provided insights into how the totality of micro-ecological practices shape exposures. The statistical analysis and modelling exercises combine observations, static sensing devices, and airflow modelling into a multimodal platform providing workers with feedback on the impact of interventions (improving ventilator positioning and opening doors) on air quality. These combined methods also provided the team with a robust sense of how interventions that seem beneficial to exposure scientists might not work for those they seek to intervene upon. These insights can be used to co-design interventions that are attuned to the reality of specific environments and specific people.

#### 4) head-to-toe interviews

**Fig. 8:**
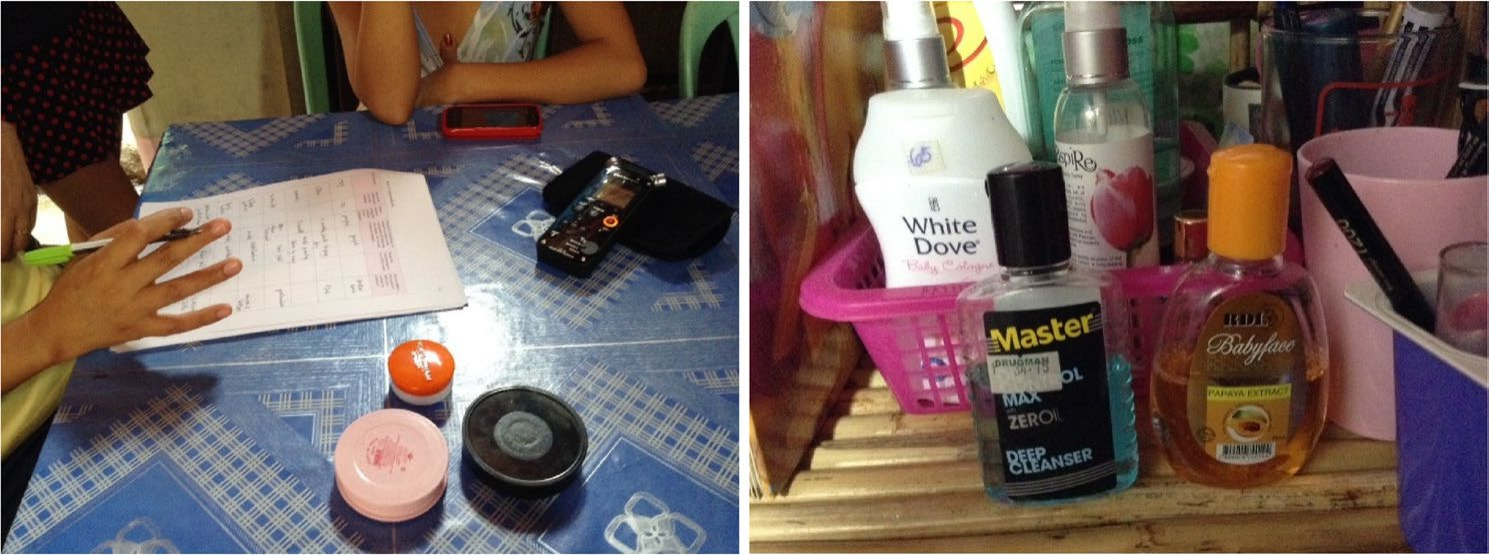
Left: interview with service-sector worker at home in Puerto Princesa, Philippines; Right: her personal care products (Photographs by Anita Hardon, 2014)

Hardon has also developed “head-to-toe interviews” for exploring how young people in the Philippines use personal care products. These interviews involve researchers visiting study participants in their homes, asking them to describe every personal care product they use from head to toe, including skin, hair, face, eyes, lips, teeth, armpits, hands, fingernails, genitalia, legs, toenails [32]. (Fig. 8). The method collects data about the kinds of products people use and why, including personal, social, economic, and affective factors (fears, aspirations, relationships) that inform their use. Rather than asking predetermined questions about specific products identified in advance, this method allows participants to determine how and what they tell researchers about which products they use and why. Integrating this methodology into exposome research integrates people’s situated practices and concerns—the arrow back—into study design, while providing quantifications of daily chemical use.

Employing this method generated extensive data about how chemical exposures accumulate in young people’s lives. Hardon’s team found that treating participants’ entire bodies systematically helped alleviate feelings of shame or stigma about sensitive chemical practices.

**Fig. 9:**
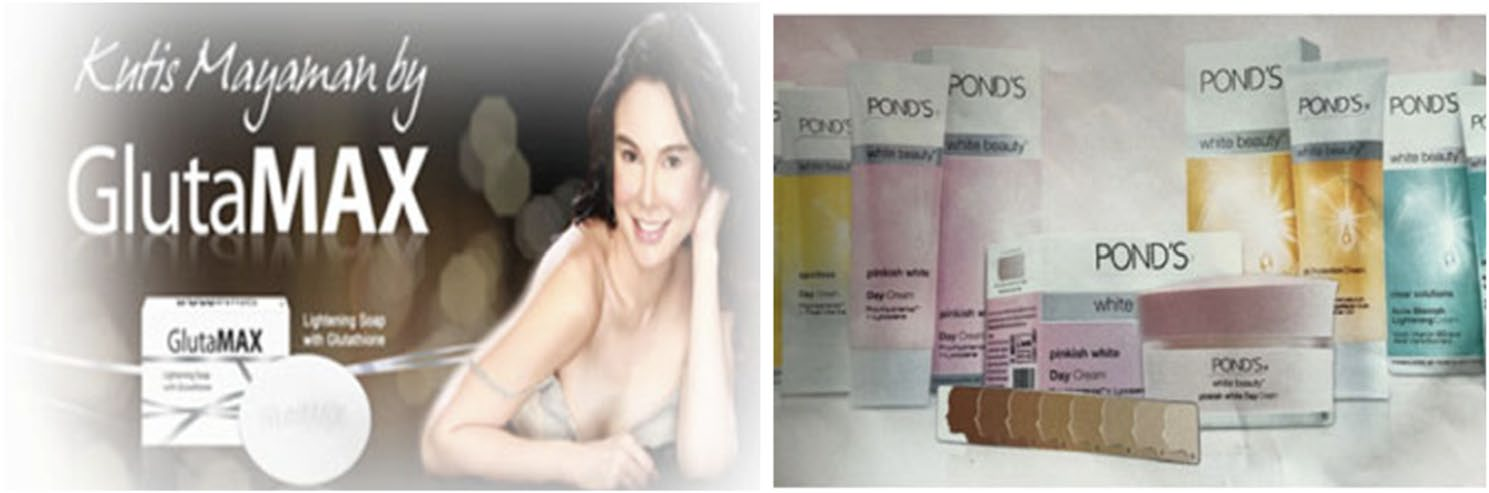
Left: Glutamax soap online advertisement, with tagline *Kutis Mayaman* (skin of the rich); Right: Ponds skin-whitening creams, marketed with a color-grading strip to “measure” their effects (Photographs by Anita Hardon, 2018)

The team also learned that young people in the Philippines routinely use multiple skin-whitening products, including soaps, scrubs, and creams, many of which contain harmful substances such as hydroquinone and mercury [33]. Participants explained they used these skin-lightening products daily because skin-lighteners increase job opportunities in the service sector (an important source of employment for young Filipinos)—which is why interventions to convince people to stop using them had very little impact. In particular, skin-lightening emerged as a key chemical practice among young indigenous (Taga-banuwa) women working in hospitality, transitioning from rural mountain life to the urban service sector in Puerto Princesa, a rural boomtown. In their remote home communities, nobody used skin-whiteners, except occasionally baby powder. In the city however, becoming hotel workers meant wearing wearing make-up. One informant told us her boss demanded she lighten her darkish skin and wear red lipstick to look pleasant for customers. Hardon’s team also observed how corporations promote skin-whitening, on TV, through social media, and on product packaging [34] suggesting that light skin is “skin of the rich” and providing users with coloring grades to measure their skin-lightening success (Fig. 9).

Drawing on data collected from head-to-toe interviews, Hardon’s team worked with study participants to design an exhibition aimed at increasing chemical literacy. The exhibition displayed popular products turned around so visitors could read their ingredients on the products’ backs to learn which contained hydroquinone or mercury. Hardon’s team discovered that, given the aggressive marketing of skin-lightening products, promoting chemical literacy alone was insufficient to reduce use, since it does not address why people use them. The “why” emerged from broader social, economic, and structural factors, including the imperative of having light(ened) skin to acquire and keep service sector jobs. Understanding this financial concern helps identify and potentially mitigate the risk pathways and health outcomes associated with exposures to skin-lightening chemicals. It is key to developing mitigation measures [35], which in this case would likely need to be structural instead of focused on individual behavior. Mitigation strategies could include regulating skin-lightening product advertisements and legislation to illegalize discrimination in the workplace based on skin color.

By first looking more broadly—from head to toe—at all the products young people used, Hardon’s team was able to describe in great detail the micro-ecological practices that shape exposures to skin-whitening products, and the complex interplay of social, economic, and environmental factors associated with these practices. Further, Hardon’s team was able to collaborate with study participants to think about effective strategies to reduce exposure.

There is a growing body of environmental health research on the use of personal care products [36–38]. These studies take stock of frequencies of and reasons for use, their harmful contents (such as formaldehyde and Formaldehyde Releasing preservatives) and their adverse reproductive health effects, elevated among women of color [38]. Anthroposomics tools complement these focused studies by being more open-ended (studying everything used head to toe), elucidating what diverse groups of users do themselves to balance benefits and harms, the arrow back [32]. Anthroposomics can help quantify micro-ecological personal care practices for inclusion in multi-omics studies that unravel the complex mechanisms through which racism, work and personal product use are related to adverse health outcomes.

#### 5) household chemical assessments

**Fig. 10:**
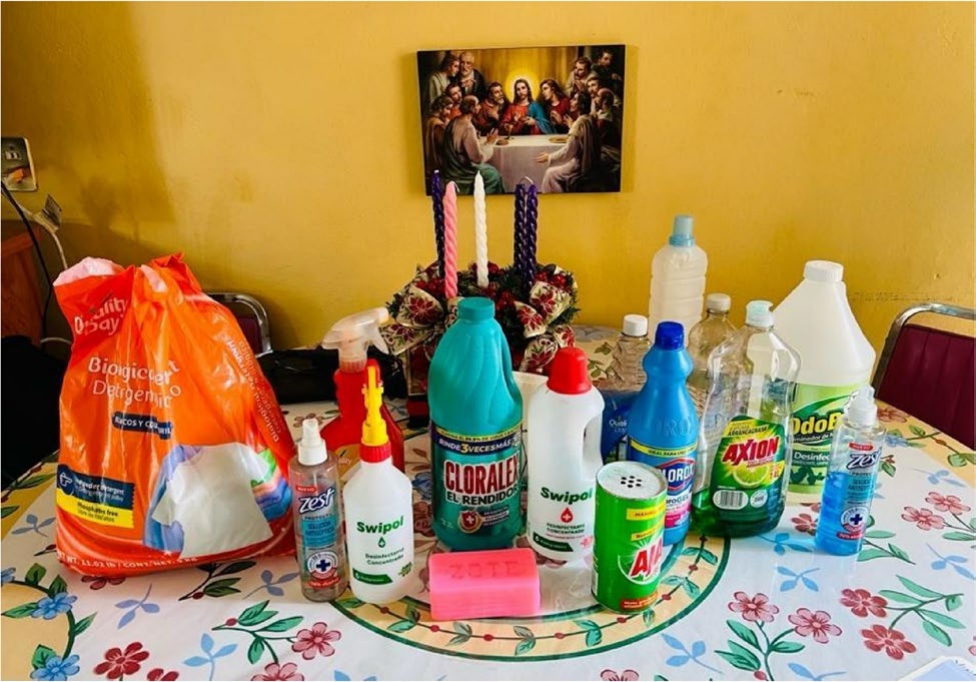
Cleaning products used in a Mexico City household (Photograph by Elizabeth F.S. Roberts, 2023)

A fifth method for collecting data about situated environmental exposures, which we have piloted in Mexico and the Philippines (Fig. 10), is Household Chemical Assessments (HCAs). Similar to head-to-toe interviews, researchers conduct home visits during which they ask residents about and catalog their household chemical product use. These include cleaning, personal care, and occupational products, like pesticides or car cleaners, as well as medication. Residents first show researchers where in the home environment they store the products (room, closet, cupboard) and then how they use the products. The researchers create a product inventory using photographs and product templates to document ingredients. During this process, researchers ask residents where they buy or otherwise acquire the products, how they make or mix them, how much of each product they use, and for what purpose(s). These assessments generate lists, frequencies, amounts, and other granular data on the chemicals to which people are routinely exposed, allowing for analysis of situated, context-specific patterns and pathways of exposure in home environments.

Piloted in just two households in the Programming Research in Obesity, Growth, Environment and Social Stressors (PROGRESS**)** study is a collaboration between Icahn School of Medicine at Mount Sinai, Harvard University and the National Institute of Public Health (INSP) cohort in Mexico City, HCAs provided the team with a total of 435 ingredients, 138 of which had unique chemical structures in the metabolomics workbench. Household chemical assessments are currently being done in a bigger sample of 60 households who are participating in the PROGRESS cohort. This non-targeted approach will allow the PROGRESS team to develop new measures and hypotheses regarding the health impacts of household chemical products by identifying previously undocumented chemical combinations in product ingredient lists.

The pilot HCA’s provided crucial data about the popularity of air-freshening products among birth cohort participants in Mexico City. Currently, public health educators in the United States tell people to avoid using air fresheners, which contain harmful chemicals, and to open their windows instead. However, during HCAs, researchers learned how Mexico City residents, who live near heavy transport zones or over faulty sewage systems continue to use air fresheners. Opening windows in this context increased exposure to unpleasant odors and potentially harmful pollutants, whereas using air fresheners improved the smell of their indoor environment. These findings highlight the “why” of exposure, the social-environmental reasons people continue to use products with potentially harmful effects. They also demonstrate that efforts to reduce the air fresheners will likely require structural changes, such as improving sewage systems that produce repellent smells and reducing air pollution.

Whereas general public health directives against using air fresheners are based on risks associated with individuals’ exposure to specific chemicals, HCAs allowed researchers to understand exposed people’s context-specific social and environmental motivations for continuing to use potentially harmful products—some arrows back. Findings like these can inform tailored, holistic strategies and interventions that account for people’s experiences in their homes and neighborhood environments, including how they manage risks associated with various kinds of exposure—keeping their windows closed, while also providing significant information for exposome research regarding little-understood indoor exposures.

## Conclusions

Collaboration with anthropologists can increase the comprehensiveness of the tools exposome researchers have available to unravel the complex interplay of environmental-social-biological factors and processes that determine not only exposures, but also their effects. Anthroposomics employs open-ended, discovery-oriented research to explore how individuals and groups sense, understand, and mitigate exposures in daily life, without relying on predefined variables or hypotheses. By directly observing the practices of exposed individuals and communities, ethnographers employing anthroposomic methods generate insights into the real-world dynamics pertaining to exposures. Our fine-grained analysis of the anthroposome reveals that affected people, who often have different priorities from environmental health researchers concerned about the risks and health impacts of specific toxicants, agentively grapple with exposures.

People living in communities with high rates of multi-source pollution, like working-class neighborhood residents in Mexico City and shoemakers in the Philippines, are often acutely aware of the constellation of exposures that comprise their exposomes. Their agency can inform the design of situated harm-reduction campaigns. Learning how and why people use air fresheners and lead-glazed ceramics in Mexico City, skin whitening creams in the Philippines, and filter the drugs they inject in the United States demonstrates how exposome research and standard health interventions may miss critical information necessary to understanding and mitigate toxic exposure.

As a rule, exposome researchers aim to identify health risks to provide evidence of them to regulators and to educate the public through top-down approaches. Anthroposomics, by contrast, builds on existing local practices and concerns. Designed to support community-based participatory research, its methods can help exposed people to identify and assess relevant toxicities based on what they encounter in their daily lives [39, 40]. Anthroposomic research provides communities and policymakers with valuable, actionable information for use in designing cost-effective, relevant hyperlocal strategies to address the health impacts of exposures. Unlike biological omics which generates ubiquitously applicable clinical interventions, our findings initially benefit local community health. For example, the Filipino shoemakers described above are at risk due to high levels of VOCs in their workshops, despite the existence of a Clean Air Act in the Philippines (RA 8749) and Philippines Department of Labor and Employment occupational health regulations that require employers to safeguard health and manage risks (including airborne hazards). The problem is therefore not a lack of regulation but lack of good governance, resulting in poor implementations of national regulations.

Anthroposomics can help scale up preventive efforts from below. For instance, we are working with community researchers in various locations to develop an anthroposomics toolkit that expands on the list of methods described in this commentary. More specifically, we are designing strategies for improving air quality in collaboration with shoemakers and workshop owners, which we hope the municipality will adopt and support financially. Our strategy involves building alliances across the city with other shoemakers to convince them of the need for interventions. Our iterative research approach will entail measuring the impact of these interventions after implementation.

We have likewise begun designing a large-scale intervention trial in Mexico with a child health development organization to reduce the use of lead-glazed *trastes de barro*. The design of this intervention incorporates insights from our anthroposomics research in in Mexico City and aims to improve upon the previous, ineffective educational intervention implemented among the ELEMENT cohort. If successful in reducing exposures and child health outcomes, this intervention could be inspire anthroposomics inquiries and intervention measures in other locations.

Anthroposomic research can scale up, converting qualitative observations into “practice” variables for multi-omics analysis, thus capturing exposure changes that other methods may miss and facilitating quantitative modeling. In two cases described above, we converted ethnographic data into binary indicators for use in regression models that analyzed the outcomes of metal and VOC exposures. These conversions demonstrated that ethnographic insights can inform exposure models, situating numeric data within real-life contexts. Our practice variables can inform the development of agent-based models, which urban exposome researchers have started recently using to examine the way people’s indoor and outdoor activities affected exposure levels [40]. By informing the design of agent-based models with practice variables, ethnographic insights can be translated into rules for causal inference modelling, enabling simulations of the impact of community-level mitigation practices [42].

Robust regulation and focused attention on the structural forces that perpetuate inequities in health [15] can best achieve environmental health. Anthroposomics, however, contributes crucial data about the complexities of people’s life circumstances, informing pragmatic harm reduction strategies that can ameliorate toxic situations [43] in locations where protective regulation does not (yet) exist. Transforming evidence into regulatory action generally takes decades [44]—and is often subject to poor implementation. Integrating anthroposomics into multi-omics exposome research can increase the utility of preventive interventions attuned to heterogeneous human–environment interactions and micro-ecological harm reduction practices. In the resource-poor settings in the Global South where we conducted our ethnographic studies, the impact of anthroposomics-informed community interventions may exceed the benefits of laboratory omics research that contributes to precision medicine at the level of the individual [9, 10]. Indeed, laboratory omics research requires extensive resources, while precision medicine, mostly implemented post exposure, likewise relies on scientific resources often unavailable in low- and middle-income settings. In contrast, by elucidating the arrows back and identifying relevant micro-ecological practices, anthroposomics can help prevent exposures and allow us to identify modifiable factors previously not on our radar. Environmental health interventions informed by anthroposomics are likely to be efficacious because they attune to what individuals comprising affected populations already practice to reduce harm.

## Data Availability

No datasets were generated or analysed during the current study.
